# The effect of outdoor air pollution on the risk of hospitalisation for bronchiolitis in infants: a systematic review

**DOI:** 10.7717/peerj.5352

**Published:** 2018-08-28

**Authors:** Charlotte King, Jamie Kirkham, Daniel Hawcutt, Ian Sinha

**Affiliations:** 1 Department of Women and Child’s Health, University of Liverpool, Liverpool, UK; 2 Department of Biostatistics, University of Liverpool, Liverpool, UK; 3 NIHR Alder Hey Clinical Research Facility, Alder Hey Children’s Hospital, Liverpool, UK; 4 Department of Respiratory Medicine, Alder Hey Children’s Hospital, Liverpool, UK

**Keywords:** Bronchiolitis, Air pollution, Hospitalisation, Systematic review

## Abstract

**Objective:**

To systematically review the evidence around the effect of ambient levels of particulate and gaseous pollutants, and the risk of hospitalisation with bronchiolitis for infants under two years of age.

**Design:**

Systematic review of observational epidemiological studies including cohort, time series, case crossover and case control study designs.

**Data sources:**

Medline, Scopus, and Web of Science searched to November 2017 with no language restrictions.

**Eligibility criteria:**

Studies investigating impact of air pollution levels on particulate pollutants (diameter <2.5 μm (PM2.5) or <10 μm (PM10) and gaseous pollutants (nitrogen dioxide (NO_2_), sulphur dioxide (SO_2_), carbon monoxide (CO), ozone (O_3_)) on hospital admission for bronchiolitis.

**Main outcome measure:**

Risk of hospitalisation from bronchiolitis.

**Results:**

Eight studies were eligible for review. Long term exposure to PM2.5 may be associated with increased risk of hospitalisation for bronchiolitis. SO_2_ may also be associated with hospitalisation, but results for other pollutants are inconsistent between studies. In three of the five studies that showed a positive association between air pollutants and hospitalisation, measured concentrations were below World Health Organization (WHO) recommended levels.

**Conclusions:**

Certain particulate and gaseous pollutants may have a clinically relevant effect on hospital admissions for bronchiolitis in children below age two years old. Large cohort or time series studies are needed to examine this possible association.

**Protocol:**

The protocol can be found at PROSPERO (CRD42017080643).

## Introduction

Bronchiolitis is a common lower respiratory infection in infancy, it is caused by respiratory syncytial virus (RSV) in 80% of cases (Jhawar, 2003). RSV, spread by droplet transmission (Leung, Kellner & Davies, 2005), causes airway inflammation (Nicolai et al., 2013), bronchial epithelial cell necrosis (Leung, Kellner & Davies, 2005), and other pathogenic effects (Sinha et al., 2015). It is a major cause of hospital admission in developed (Green et al., 2016; Hasegawa et al., 2013; Shay et al., 1999) and developing countries (Berman, 1991; Robertson et al., 2004) worldwide. In England, rates of hospital admission for infants with bronchiolitis have increased since the 1980s, particularly in industrialised areas (Green et al., 2016). Risk factors for severe disease requiring hospitalisation include those that affect structural and functional lung development, or generate airway inflammation, including prematurity (García et al., 2010), low birth weight (Lanari et al., 2002), cardiac abnormalities (Purcell & Fergie, 2004), and exposure to tobacco smoke (Semple et al., 2011).

Exposure to air pollutants in early childhood also affects pulmonary function tests, and airway inflammation (Gotschi et al., 2008; Schultz et al., 2012). Relative functional and anatomical immaturity of an infant’s respiratory and immune systems, in addition to their higher tidal volume per unit body weight, may render them particularly susceptible to the adverse effects of air pollutants (Braga et al., 2001; Schwartz, 2004).

There is increasing awareness that air pollution contributes to respiratory morbidity (Curtis et al., 2006; UNICEF, 2016) and mortality (Global Burden of Disease 2016 Risk Factors Collaborators, 2017; Marchal et al., 2012), including increased risk of asthma exacerbations (Orellano et al., 2017) and acute lower respiratory infection in children (Mehta et al., 2013). Air pollutants implicated in respiratory morbidity, their major sources (World Health Organization (WHO), 2006; UNICEF, 2016) and maximum recommended World Health Organization (WHO) ambient levels (World Health Organization (WHO), 2000, 2006) are listed in Table 1. The air pollutants implicated are particulate matter, which is subdivided depending on particle size as either less than 2.5 μm (PM2.5) or less than 10 μm (PM10), nitrogen dioxide (NO_2_), sulfur dioxide (SO_2_), carbon monoxide (CO), and ozone (O_3_).

**Table 1:** Air pollutants that affect the respiratory system, their major sources, and maximum mean levels recommended by WHO.

	Source	WHO ambient level
Particulate matter diameter <2.5 μm (PM2.5)	Combustion sources	10 μg/m^3^ annual mean 25 μg/m^3^ 24 h mean
Particulate matter diameter <10 μm (PM10)	Mechanical processes such as construction activities, road dust re-suspension and wind	20 μg/m^3^ annual mean 50 μg/m^3^ 24 h mean
Nitrogen Dioxide (NO_2_)	Fuel emission and combustion related pollution i.e. road traffic	40 μg/m^3^ annual mean 200 μg/m^3^ 1 h mean
Sulphur Dioxide (SO_2_)	Fossil fuel combustion at industrial plants and other industrial facilities	20 μg/m^3^ 24 h mean 500 μg/m^3^ 10 min mean
Carbon Monoxide (CO)	Fossil fuel emission from cars, trucks and other vehicles	10 mg/m^3^ 8 h mean
Ozone (O_3_)	Photochemical reactions in the presence of sunlight and oxides or VOCs	100 μg/m^3^ 8 h mean

We aimed to systematically review observational studies which examined the impact of ambient levels of both particulate and gaseous pollutants on the risk of hospitalisation with a clinical or microbiological diagnosis of bronchiolitis in infants.

## Methods

The protocol for our review was registered on PROSPERO (CRD42017080643). Two investigators (Charlotte King and Ian Sinha) independently performed the initial screening of titles and abstracts, analysed full text reports for eligibility, extracted data, and evaluated study quality according to the steps of the PRISMA statement (Liberati et al., 2009). Disagreements were discussed between the two reviewers (Charlotte King and Ian Sinha) to reach an agreement, a third reviewer (DH) then assessed if agreement was not made.

### Information sources and search strategy

We individually searched online databases MEDLINE, SCOPUS and Web of Science for terms related to ‘bronchiolitis’, ‘air pollution’, ‘particulate matter’, ‘nitrogen dioxide’, ‘sulphur dioxide’, ‘carbon monoxide’, ‘ozone’, and ‘infants’ (see File S1 for detailed search strategy).

### Inclusion criteria and study selection

We included cohort, time series, case crossovers or case control study designs (based on previous methodology by Shah et al. (2015)) that evaluated the impact of air pollution levels (PM2.5, PM10, NO_2_, SO_2_, CO, O_3_) on the pre-specified primary outcome (risk of hospitalisation with bronchiolitis, this is classified as a hospital admission only not including emergency department visits). Secondary outcomes were the risk of emergency department visits, unscheduled primary care visits, and critical care admission. Studies were included that evaluated exposure to air pollutants at any time period (lag) before hospitalisation occurred and were categorised as acute (less than 7 days), sub-chronic (1 month prior to hospitalisation), or lifetime exposure (average daily exposure from birth to hospitalisation). We specified that the primary pollutants of interest would be particulate matter and nitrogen dioxide as these are from the most common sources of pollution in urban areas (UNICEF, 2016).

There were no language restrictions. We excluded studies that evaluated the impact of air pollution on more than one respiratory illness if data for bronchiolitis were not presented separately. We also excluded studies examining temporal associations between air pollution levels and the number of hospitalisations for bronchiolitis in a particular hospital or region.

### Assessment of quality of studies

The quality of case-control and cohort studies was evaluated using the Newcastle Ottawa quality assessment tool (Wells et al., 2015), and in addition the following specific methodological features of all included studies were examined.

#### Selection bias and additional quality criteria

Studies were considered to have low risk of selection bias if consecutive cases of hospital admission for bronchiolitis were included, and these were identified from health records rather than parental recall. We classed as higher quality those studies in which the case definition of bronchiolitis was based on the International Classification of Disease (ICD 9) criteria (World Health Organization, 1978) (code 466.1, due to RSV or other infectious organisms), and whether clinical diagnosis was supplemented by microbiological testing. Also, studies were classified as higher quality if infants were less than two years old based on guidance by the National Institute for Health and Care Excellence (NICE) (Ralston et al., 2014).

#### Exposure assessment

From each study, we evaluated the reported methodology with which levels of air pollutants were measured, regarding frequency of monitoring, methodology of data collection and proximity of stations to participants. Studies were considered more methodologically robust if pollutants were measured daily, using standardised techniques, and monitors were placed within ten miles of the hospitals or residences.

#### Adjustment for confounders

Adjustment for meteorological confounders, socioeconomic status, age, and other clinical risk factors were examined in each study. Studies were considered to be at low risk of bias if they adjusted for at least two of these types of confounders.

### Data extraction and statistical analysis

From eligible studies, we extracted data around sample size, odds ratio (OR) or relative risk of hospital admission for the stated air pollutant concentrations. We desired to only meta-analyse results from cohort studies, as they provide the strongest observational evidence in the absence of RCTs. Case crossover studies allow for case events to act as their own controls, with excess risk evaluated using conditional logistic regression (Maclure, 1991), thus adjust for age as a confounding variable. Time series studies use counts with analysis done using log linear regression models, adjusting for confounding variables such as the weather (Fung et al., 2003). Time series, case crossover studies and case control studies are reported descriptively, and results presented on forest plots without overall synthesis. This was undertaken due to the high levels of methodological heterogeneity expected between studies. For each study, we compared the mean ambient pollutant value with the recommended level by WHO (2000, 2006).

### Grading of evidence

For acute, sub-chronic, and lifetime exposure to each pollutant, we formulated conclusions and graded evidence according to a strategy based on recommendations from the GRADE working group (Schünemann et al., 2013), such that each conclusion would be based on low-, moderate- or high-quality evidence as judged by two reviewers (Ian Sinha and Charlotte King). Evidence was graded as low to begin with, as we only included observational studies, and was downgraded one level if there was only one study. Evidence was graded down if there were any studies in the analysis with one or more methodological limitations outlined in the quality assessment domains above and graded up one if there were no methodological flaws across the studies relevant to that analysis. We considered downgrading one level for inconsistency if there were no overlapping confidence intervals between studies, if the point estimate for OR across studies was either wide in variance across studies, or if the results were conflicting. We did not include criteria around indirectness this was covered in the quality assessment process. For imprecision, we graded down if there were less than 5,000 infants in the studies and graded up one if there were more than 20,000. We did not incorporate formal assessment of publication bias as there were too few studies to do this robustly.

## Results

**Figure 1:**
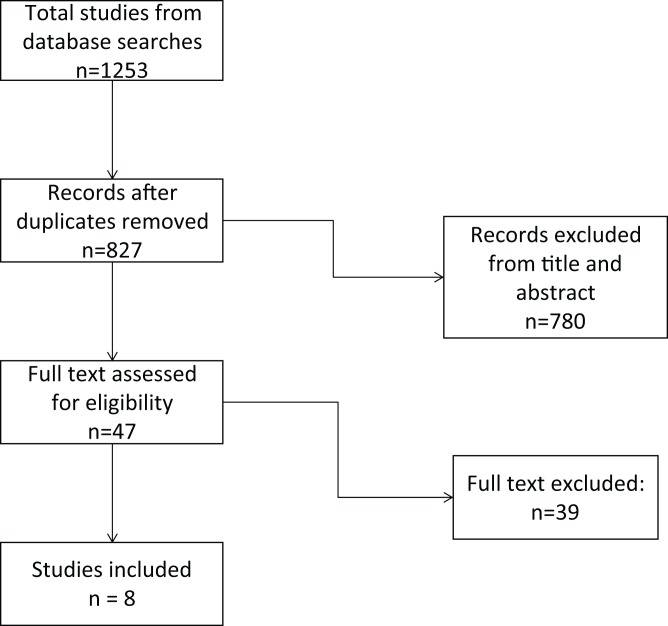
PRISMA flow diagram.

Eight studies (including four case control studies and four case crossover studies Abdul Rahman et al., 2017; Girguis et al., 2017; Karr et al., 2004, 2006, 2007, 2009a, 2009b; Segala et al., 2008) were eligible for our review. The review flowchart is shown in Fig. 1, and the reasons individual studies were excluded are summarised in File S2.

Six of the studies were from overlapping research groups in North America (Girguis et al., 2017; Karr et al., 2004, 2006, 2007, 2009a, 2009b), one from France (Segala et al., 2008) and one from Malaysia (Abdul Rahman et al., 2017) a developing country. The study characteristics are summarised in Table 2. Of the eight studies, six were classed as being of low risk of bias with regards selection of participants, evaluation of air pollution, and for adjustment for confounding factors in their analysis. The study characteristics are summarised in Table 2, and the quality assessments in Table 3.

**Table 2:** Characteristics of included studies.

Study	Study design	Years conducted	Country (Region)	Bronchiolitis definition	Population	Population size	Lag exposure	Adjusted for confounders	Pollutants measured*
Karr et al. (2009a)	Case control	1999–2002	Canada (British Columbia)	ICD 9	Singleton children aged 2–12 months	11,675	Lifetime and 1 month before	Adjusted for infant sex, gestational age, First Nation status, parity, maternal age, maternal smoking during pregnancy, maternal initiation of breastfeeding at birth, income (quintile census), maternal education (quartile census). Cases and controls are matched on date of birth	PM2.5, PM10, NO_2_, SO_2_, CO, O_3_
Karr et al. (2009b)	Case control	1997–2003	United States (Washington State)	ICD 9	Three weeks to one year	3,124	Lifetime, 30 day average and 7 day average (PM2.5 only)	Maternal education, mother’s self-reported smoking during pregnancy and infant race/ethnicity	PM2.5, NO_2_
Karr et al. (2006)	Case crossover	1995–2000	United States (California)	ICD 9	Three weeks to one year	19,109	Lag 1–2 and Lag 3–5 days for PM2.5, Lag 1 and 4 days for NO_2_, CO	Day of week (PM2.5 only), mean daily temperature, mean daily humidity	PM2.5, NO_2_, CO
Karr et al. (2004)	Case crossover	1995–2000	United States (California)	Not stated	Three weeks to one year	22,365	Lag 1–2, Lag 3–5	Not stated	PM2.5, NO_2_, CO
Girguis et al. (2017)	Case control	2001–2008	United States (Massachusetts)	ICD 9	Three weeks to less than 12 months	19,374	Lifetime	High risk pregnancy, maternal age, birthweight, smoking during pregnancy, maternal education, adequacy of prenatal care, parity, income and insurance type. Matched on date of birth (± 6 days) and gestational week	PM2.5
Abdul Rahman et al. (2017)	Case crossover	2006–2010	Malaysia (Klang Valley)	Not stated	Not stated	5,779	Lifetime	Not stated	PM10, CO, O_3_, NO_2_
Karr et al. (2007)	Case control	1995–2000	United States (California)	ICD 9	Three weeks to one year	18,595	Chronic and sub-chronic	Gender, ethnicity (Hispanic vs. not Hispanic), insurance category (medical, private/health maintenance organization/preferred provider organization, other), mother’s highest level of education (0, 1–6, 7–12, or 13 years), any lung disease (chronic lung disease and pulmonary anomalies, including congenital diaphragmatic hernia), any cardiac anomalies, daily mean temperature, and daily mean humidity	PM2.5, NO_2_, O_3_, CO
Segala et al. (2008)	Case crossover	1997–2001	France (Paris)	Respiratory dyspnea and/or sibilants and wheezing for children	Less than three years	16,588	Lag 0–1, lag 0–4	Public holidays, holidays and weather variables	PM10, NO_2_, SO_2_

**Note:**

* PM2.5, particulate matter diameter <2.5 μm; PM10, particulate matter diameter <10 μm; NO_2_, nitrogen dioxide; SO_2_, Sulphur dioxide; CO, carbon monoxide; O_3_, ozone.

**Table 3:** Risk of bias assessment of included studies.

Study	Study design	Selection of participants	Evaluation of exposure	Consideration of confounding factors	Newcastle Ottawa score
Karr et al. (2009a)	Case control	Low	Low	Low	7
Karr et al. (2009b)	Case control	Low	Low	Low	7
Karr et al. (2006)	Case crossover	Low	Low	Low	N/A
Karr et al. (2004)	Case crossover	Unclear*	Unclear*	Unclear*	N/A
Girguis et al. (2017)	Case control	Low**	Low	Low	8
Abdul Rahman et al. (2017)***	Case crossover	High	High	High	N/A
Karr et al. (2007)	Case control	Low	Low	Low	7
Segala et al. (2008)	Case crossover	Low	Low	Low	N/A

**Notes:**

* Unclear as conference abstract.

** In this study, hospital admissions, observational stays, and ED visits were combined into one outcome (‘clinical encounter’) but data for hospitalisations only were reported separately.

*** Unclear risk of bias for selection as although all admissions were included, definition of bronchiolitis is not stated; High risk of bias for exposure evaluation based on large distance between measurement stations; no adjustment for confounding factors.

### Association between air pollution and risk of hospitalisation for bronchiolitis

**Figure 2:**
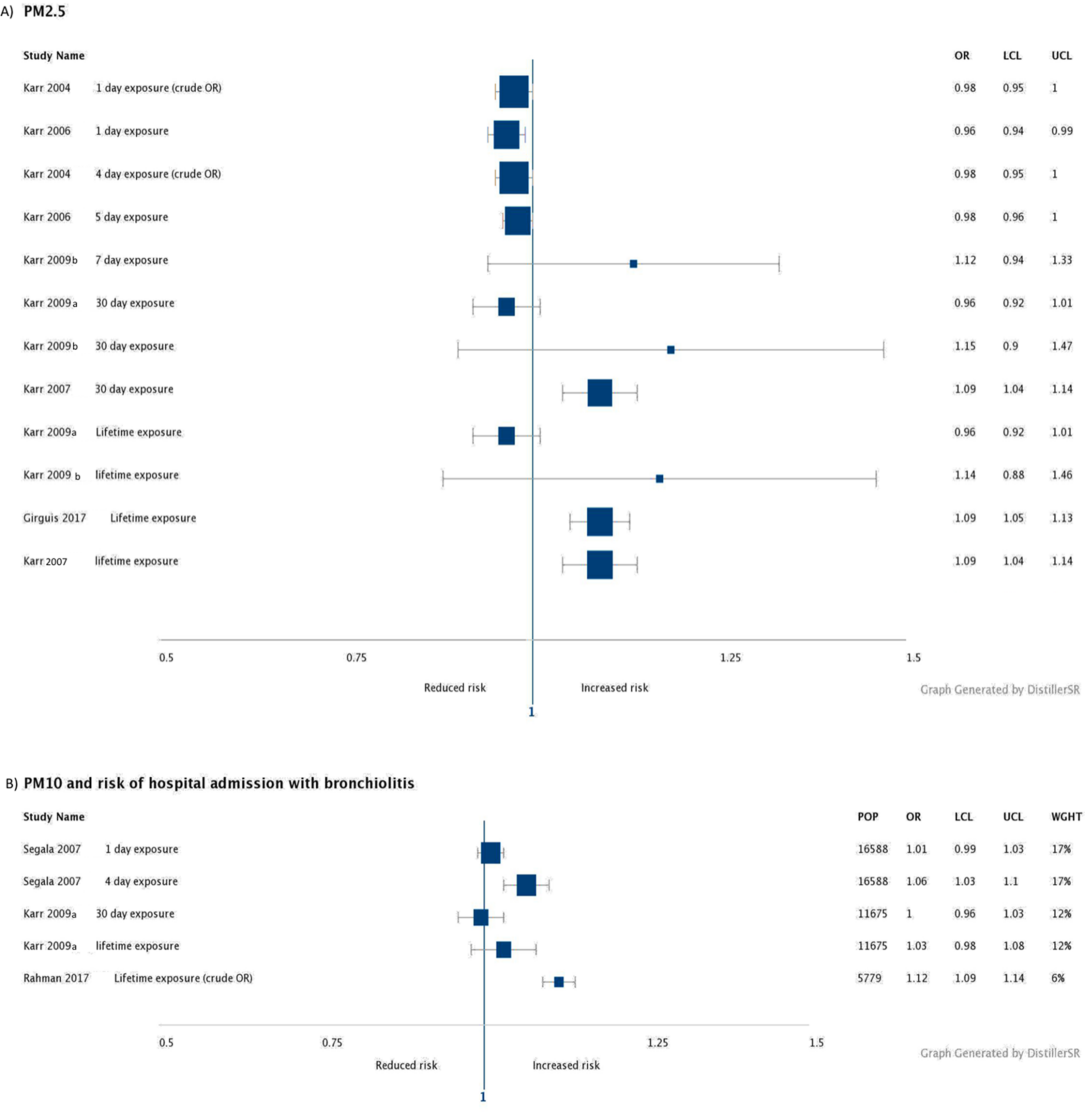
Forest plots of particulate pollutants. (A) PM2.5 forest plot. (B) PM10 forest plot.

**Figure 3:**
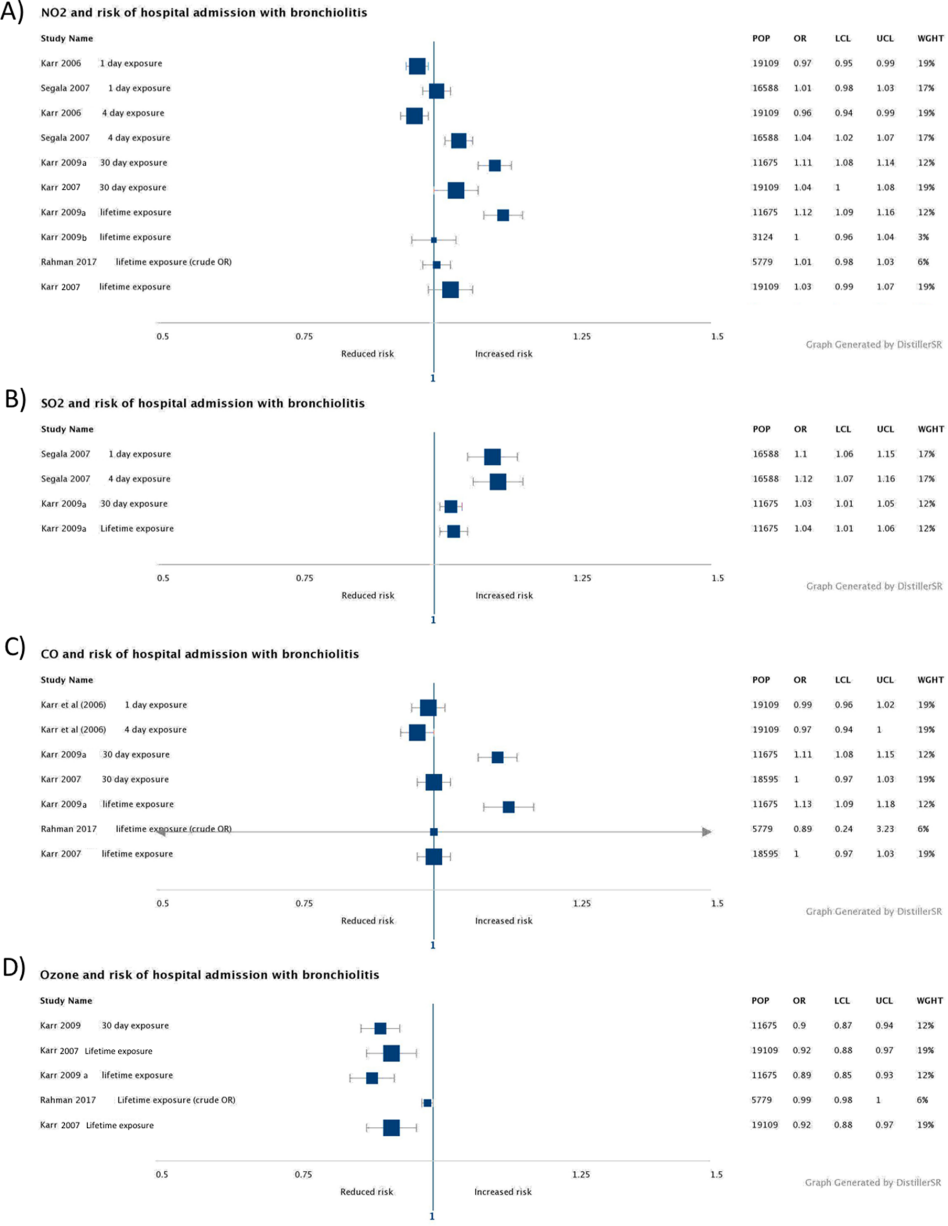
Forest plots of gaseous pollutants. (A) NO_2_ forest plot. (B) SO_2_ forest plot. (C) CO forest plot. (D) O_3_ forest plot.

The results from the included studies are summarised below and shown in Figs. 2 and 3, see File S3 for detailed results. The summary evidence and GRADE assessments are summarised in File S4.

### Particulate pollutants

Based on moderate quality evidence (File S4), acute exposure to PM2.5 does not seem to increase risk of hospitalisation (Fig. 2). Two studies (Karr et al., 2004, 2006) found no increased risk of hospitalisation with acute exposure to PM2.5. Sub-chronic effects are unclear, but lifetime exposure may increase risk of hospitalisation (Fig. 2). Two studies found no increased risk of hospitalisation with sub chronic or lifetime exposure (Karr et al., 2009a, 2009b) but two studies did find an increased risk with 30 day exposure (OR 1.09 [1.04–1.14]) and lifetime exposure (OR 1.09 [1.04–1.14] Karr et al., 2007 and 1.09 [1.05–1.13] Girguis et al., 2017).

The evidence around PM10 is of lower quality (File S4). Acute and lifetime effects of PM10 on hospitalisation with bronchiolitis are unclear, but sub chronic exposure does not seem to be associated with increased risk (Fig. 2). One study (Segala et al., 2008) found association between PM10 and risk of hospital admission at a lag of 0–4 days (OR 1.06 [1.03–1.10]), but not at a lag of 0–1 days. Of the two studies which measured longer term effects of PM10, one (Karr et al., 2009a) found no association, but one (Abdul Rahman et al., 2017) found a statistically significant association with lifetime exposure (OR 1.115 [1.093–1.138]).

### Gaseous pollutants

The association between exposure length of gaseous pollutant and risk of hospitalisation admission varied between pollutants, and results were inconsistent across studies.

Based on moderate quality evidence (File S4), the acute, sub-chronic, and lifetime effects of NO_2_ are unclear, although longer term exposure may be associated with increased risk of admission for bronchiolitis (Fig. 3). Two studies found no increased risk of acute exposure (Karr et al., 2004, 2006), and one found a statistically significant association at a lag of 0–4 days (OR 1.04 [1.02–1.07]), but not 0–1 days (Segala et al., 2008). Three studies (Abdul Rahman et al., 2017; Karr et al., 2007, 2009b) found no association with risk of hospitalisation admission, but one (Karr et al., 2009a) found statistically significant association with sub-chronic (OR 1.11 [1.08–1.14]) and lifetime exposure (OR 1.12 [1.09–1.16]).

For SO_2_, the strength of this evidence was graded as low (File S4). The results of two studies suggest that acute, sub-chronic, and lifetime exposure to SO_2_ may be associated with increased risk of hospitalisation (Fig. 3). One study (Segala et al., 2008) examined the acute effects and found statistically significant associations at a lag of 0–4 days (OR 1.12 [1.07–1.16]) and 0–1 days (OR 1.10 [1.06–1.15]). One study (Karr et al., 2009a) assessed longer term exposure and found a statistically significant association with risk of hospitalisation for lifetime exposure (OR 1.04 [1.01–1.06]) and sub-chronic exposure (OR 1.03 [1.01–1.05]).

Based on low-quality evidence from two studies (Karr et al., 2004, 2006), CO does not seem to have acute effects on the risk of hospitalisation for bronchiolitis (Fig. 3). Based on moderate quality evidence for sub-chronic effects, and low-quality evidence for lifetime exposure effects, the risks of hospitalisation for bronchiolitis in relation to longer term exposure to CO is unclear (Fig. 3). Two studies found no association with risk of hospitalisation (Abdul Rahman et al., 2017; Karr et al., 2007), but one study (Karr et al., 2009a) found a statistically significant association with lifetime (OR 1.13 [1.09–1.18]) and sub-chronic exposure (OR 1.11 [1.08–1.15]).

Three studies assessed long-term effects of ozone but none evaluated acute exposure. The quality of evidence for sub-chronic as moderate, but low for lifetime exposure (File S4). Most studies showed a reduction in the risk of admission associated with ozone exposure (Fig. 3). One study (Abdul Rahman et al., 2017) found no association between ozone levels and risk of hospitalisation for bronchiolitis. Two studies however found a statistically significant decrease in hospitalisation risk with long-term exposure to ozone, one with sub-chronic (OR 0.90 [0.87–0.94]) and lifetime (OR 0.89 [0.85–0.93]) exposure (Karr et al., 2007, 2009a) and one with lifetime exposure (OR 0.92 [0.88–0.97], Karr et al., 2007, 2009a).

### Secondary outcomes

One case crossover study (Segala et al., 2008) examined the acute effect of PM10, NO_2_ and SO_2_ on risk of unscheduled consultation for bronchiolitis. One case control study (Girguis et al., 2017) evaluated lifetime exposure of PM2.5 on risk of clinical encounter for bronchiolitis and found no association.

A statistically significant association was found at a lag of 0–4 days for PM10 (OR 1.06 [1.04–1.08]), NO_2_ (OR 1.03[1.02–1.05]), and for SO_2_ (OR 1.12[1.09–1.15]), which was also statistically significant for a lag of 0–1 days (OR 1.08[1.06–1.11]).

### Comparison between effect of air pollution and WHO recommended guidelines

Of the eight included studies, five reported that one or more pollutants was associated with an increased risk of hospitalisation for bronchiolitis (Abdul Rahman et al., 2017; Girguis et al., 2017; Karr et al., 2007, 2009a; Segala et al., 2008). Of these, three measured mean levels of air pollutants below the WHO recommendations (Girguis et al., 2017; Karr et al., 2009a; Segala et al., 2008). Two studies (Karr et al., 2009a; Girguis et al., 2017) found statistically significant associations between PM2.5 and risk of hospitalisation from bronchiolitis, at mean levels below WHO recommendations, and one (Segala et al., 2008) had similar findings for PM10.

## Discussion

This is the first systematic review analysing the effect of exposure to ambient air pollution on the risk of hospital admission with bronchiolitis. Although findings are inconsistent across studies a suggested association with longer term and lifetime exposure to particulate matter on the risk of hospitalisation for bronchiolitis is seen. Acute exposure to NO_2_ and SO_2_, may also be associated with increased risk of hospitalisation. In some studies (Girguis et al., 2017; Karr et al., 2009a, 2009b), hospitalisation with bronchiolitis increased despite measured levels of the gaseous pollutants being lower than the maximum concentrations recommended in WHO guidelines.

Ozone, is known to be a unique air pollutant and is often peaked during the hot season when RSV epidemics are low. Thus, would not seem to affect risk of hospitalisation with bronchiolitis. However, the decreased risk does not necessarily mean that ozone is a protective factor. It could be that due to the increase in temperature the photochemical reaction producing ozone is increased in the high season (Monks et al., 2014), or that in the winter months, other pollutants confound the effects of ozone, multipollutant modelling is one way to assess this (Karr et al., 2007).

It is biologically plausible that air pollutants might increase the likelihood of severe bronchiolitis, because of known effects on lung function (Gotschi et al., 2008; Yu et al., 2001) and airway inflammation (Barraza-Villarreal et al., 2008). In systematic reviews of epidemiological studies, risk of asthma exacerbations in children was increased with exposure to particulate pollutants, O_3_, SO_2_ and NO_2_ (Orellano et al., 2017), and the risk of acute lower respiratory infections is associated with PM2.5 exposure (Mehta et al., 2013). The possible differences between pollutants with regards to the chronicity of their association with hospitalisation for bronchiolitis may reflect different pathogenic processes. PM2.5 and PM10 may have a more chronic pro-inflammatory effect (Calderón-Garcidueñas et al., 2003), while NO_2_ and SO_2_ may be associated with more acute damage to airways (Chen et al., 2007), but further work is required to better understand the in vivo pathogenic effects of these pollutants in the airways of infants and children (Aguilera et al., 2013).

Maximum levels of air pollutants in current WHO air quality guidelines may not be sufficiently low to protect infants, who may be particularly vulnerable to their harmful effects (Salvi, 2007). In a study that examined adverse effects of air pollution exposure on children’s health, infants younger than two years of age were most susceptible to the health effects of air pollutants, particularly NO_2_, SO_2_, and PM10 (Braga et al., 2001). In context of increased urbanisation worldwide (Landrigan et al., 2018) it is important that the impact of air pollution on infants is considered when writing guidelines about air quality. Currently, 98% of cities in low and middle income countries with populations greater than 100,000 people, and 56% of such cities in high income countries, demonstrate air pollution levels above the WHO guidelines (World Health Organization (WHO), 2016).

This review was conducted in a systematic manner, but the validity of the conclusions is hampered by variation between studies. From current evidence, it is difficult to estimate the proportion of cases of hospitalisation from bronchiolitis that may be attributable to air pollution but given the ubiquity of this infection even the modest associations identified in this review are likely to have a substantial impact on morbidity and global burden of illness. Seasonality is a known to affect the variability of air pollution, with the majority of studies accounting for temperature and humidity along with matching within the same time period for time-series and case crossover studies to limit this confounder (Girguis et al., 2017; Karr et al., 2006, 2007, 2009b; Segala et al., 2008).

Although this systematic review only analysed the effects of air pollution to risk of hospitalisations after birth, emerging evidence suggests an association between antenatal air pollution exposure and low birthweight (Smith et al., 2017) which may also affect risk of severe bronchiolitis. In a Spanish cohort study, NO_2_ exposure in the second trimester was positively associated with an increased risk of doctor diagnosed lower respiratory tract infection, with 98% of the diagnosis being classified as bronchiolitis or bronchitis (Aguilera et al., 2013), and also highlights the possibility of antenatal exposure to air pollutants being a risk factor for bronchiolitis. A recent study, published after the search period, has further highlighted positive associations between traffic related pollutants, PM2.5, CO, and nitrogen oxides (this includes NO_2_), and bronchiolitis clinical encounters (Kennedy et al., 2018). This further supports that air pollution may have an association with increased risk of bronchiolitis hospital admissions.

The results of the included studies were unable to be synthesised as no studies were identified that utilised a cohort design. One source of imprecision is that the diagnosis of bronchiolitis, even when made according to standardised definition, relies upon the subjective judgements by individual clinicians. Variation in the age definition between the studies may have resulted in viral wheeze being misclassified as bronchiolitis, particularly when including over the age of one year (Abdul Rahman et al., 2017; Segala et al., 2008). Differences in the way in which air pollution was measured was observed, and the confounding factors that were considered in the analyses of the studies. As expected, crude odds ratios that showed statistical significance were found (Girguis et al., 2017; Karr et al., 2009a), but adjusted ORs did not, and this highlights the importance of considering confounding factors in observational studies. It was noted that studies measuring exposure to more than one pollutant did not describe a pre-specified primary analysis with regards to clinical outcome, pollutant, and lag time. It is possible, therefore, that individual studies may be at risk of selective outcome reporting, a practice that is commonplace in RCTs (Dwan et al., 2013). International consensus, around potential confounding factors and a core outcome set (Kirkham, Clarke & Williamson, 2017; Sinha et al., 2012) to measure and report in observational studies of air pollution, may help reduce these problems.

## Conclusion

This review suggests an association between different air pollutants and risk of hospitalisation for bronchiolitis, particularly with particulate matter, NO_2_ and SO_2_ exposure. There is a need for a multicentre cohort or time series to examine this possible association, and these would be strengthened by development of standardised methodological approaches. Revision of international recommendations around air quality is warranted and this should incorporate specific consideration around the impact of outdoor air pollution on infants.

## Supplemental Information

10.7717/peerj.5352/supp-1 Supplemental Information 1 PRISMA checklist.

10.7717/peerj.5352/supp-2 Supplemental Information 2 Supplementary File S1: Search Strategy.

10.7717/peerj.5352/supp-3 Supplemental Information 3 Supplementary File S2: Table of excluded studies.

10.7717/peerj.5352/supp-4 Supplemental Information 4 Supplementary File S3: Results table of included studies.

10.7717/peerj.5352/supp-5 Supplemental Information 5 Supplementary File S4: Grade assessments.

10.7717/peerj.5352/supp-6 Supplemental Information 6 Systematic review/meta-analysis justification.

## Additional Information and Declarations

### Data Availability

The following information was supplied regarding data availability: The research in this article did not generate any data or code as it is a systematic review.
