# Supplementary material for: The effect of outdoor air pollution on the risk of hospitalisation for bronchiolitis in infants: a systematic review
Source: PeerJ. 2018 Aug 28;6:e5352. doi: 10.7717/peerj.5352 (PMC6118201; doi:10.7717/peerj.5352)
Supplement: Supplemental Information 2 [file peerj-06-5352-s002.pdf]

S1 – Detailed Search Strategy

Web of Science

1. **TOPIC:** (baby) *OR* **TOPIC:** (infan*) *OR* **TOPIC:** (pediatric*) *OR* **TOPIC:** (paediatric*) *OR***TOPIC:** (peadiatric*) *AND* **TOPIC:** (child*) *AND* **TOPIC:** (toddler*) *AND* **TOPIC:** (pre-school*)
   Indexes=SCI-EXPANDED, SSCI, A&HCI, CPCI-S, CPCI-SSH, BKCI-S, BKCI-SSH, ESCI, CCR-EXPANDED, IC Timespan=All years
2. **TOPIC:** (Bronchiolitis) *OR* **TOPIC:** (RSV) *OR* **TOPIC:** ("respiratory syncytial virus")
   Indexes=SCI-EXPANDED, SSCI, A&HCI, CPCI-S, CPCI-SSH, BKCI-S, BKCI-SSH, ESCI, CCR-EXPANDED, IC Timespan=All years
3. (sulfur dioxide) *OR* **TOPIC:** (SO2) *OR* **TOPIC:** (sulphur dioxide)
   Indexes=SCI-EXPANDED, SSCI, A&HCI, CPCI-S, CPCI-SSH, BKCI-S, BKCI-SSH, ESCI, CCR-EXPANDED, IC Timespan=All years
4. **TOPIC:** (nitrogen dioxide) *OR* **TOPIC:** (NO2)
   Indexes=SCI-EXPANDED, SSCI, A&HCI, CPCI-S, CPCI-SSH, BKCI-S, BKCI-SSH, ESCI, CCR-EXPANDED, IC Timespan=All years
5. (carbon monoxide) *OR* **TOPIC:** (CO)
   Indexes=SCI-EXPANDED, SSCI, A&HCI, CPCI-S, CPCI-SSH, BKCI-S, BKCI-SSH, ESCI, CCR-EXPANDED, IC Timespan=All years
6. (ozone) *OR* **TOPIC:** (O3)
   Indexes=SCI-EXPANDED, SSCI, A&HCI, CPCI-S, CPCI-SSH, BKCI-S, BKCI-SSH, ESCI, CCR-EXPANDED, IC Timespan=All years
7. (particulate matter) *OR* **TOPIC:** (PM)
   Indexes=SCI-EXPANDED, SSCI, A&HCI, CPCI-S, CPCI-SSH, BKCI-S, BKCI-SSH, ESCI, CCR-EXPANDED, IC Timespan=All years
8. (TS=(air OR ambient OR atmospher* OR outdoor))
   Indexes=SCI-EXPANDED, SSCI, A&HCI, CPCI-S, CPCI-SSH, BKCI-S, BKCI-SSH, ESCI, CCR-EXPANDED, IC Timespan=All years
9. (TS=(pollution OR quality))
   Indexes=SCI-EXPANDED, SSCI, A&HCI, CPCI-S, CPCI-SSH, BKCI-S, BKCI-SSH, ESCI, CCR-EXPANDED, IC Timespan=All years
10. #8 AND #9
    Indexes=SCI-EXPANDED, SSCI, A&HCI, CPCI-S, CPCI-SSH, BKCI-S, BKCI-SSH, ESCI, CCR-EXPANDED, IC Timespan=All years
11. #3 OR #4 OR #5 OR #6 OR #7 OR #10
    Indexes=SCI-EXPANDED, SSCI, A&HCI, CPCI-S, CPCI-SSH, BKCI-S, BKCI-SSH, ESCI, CCR-EXPANDED, IC Timespan=All years
12. #11 AND #1 AND #2
    Indexes=SCI-EXPANDED, SSCI, A&HCI, CPCI-S, CPCI-SSH, BKCI-S, BKCI-SSH, ESCI, CCR-EXPANDED, IC Timespan=All years

Scopus

1. TITLE-ABS-KEYS (baby OR infan* OR pediatric* OR paediatric* OR peadiatric* OR child* OR toddler* OR pre-school*)

2. TITLE-ABS-KEYS (bronchiolitis or RSV or "respiratory syncytial virus")
3. TITLE-ABS-KEYS (sulfur AND dioxide OR SO2)
4. TITLE-ABS-KEYS (nitrogren AND dioxide OR NO2)
5. TITLE-ABS-KEYS (ozone OR O3)
6. TITLE-ABS-KEYS (carbon AND monoxide OR CO)
7. TITLE-ABS-KEYS (particulate AND matter OR PM*)
8. TITLE-ABS-KEYS (air OR ambient OR atmospher* OR outdoor)
9. TITLE-ABS-KEYS (pollution OR quality)
10. #8 AND #9
11. #10 OR #7 OR #6 OR #5 OR #4 OR #3
12. #11 AND #1 AND #2

MEDLINE via OVID

1. Sulfur dioxide/ or (sulfur dioxide or SO2 or sulphur dioxide).mp.
2. (nitrogen dioxide or NO2).mp. or Nitrogen Dioxide/
3. (ozone or O3).mp. or ozone/
4. (carbon monoxide or CO).mp. or Carbon Monoxide/
5. (particulate matter or PM*).mp. or Particulate Matter/
6. ((air or ambient or atmospher* or outdoor) adj1 (pollution or quality)).mp.
7. (bronchiolitis or RSV or "respiratory syncytial virus").mp.
8. (baby or infan* or pediatric* or paediatric* or peadiatric* or child* or toddler* or pre-school*).mp.
9. or/1-6
10. 7 and 8 and 9
