# Supplementary material for: The effect of outdoor air pollution on the risk of hospitalisation for bronchiolitis in infants: a systematic review
Source: PeerJ. 2018 Aug 28;6:e5352. doi: 10.7717/peerj.5352 (PMC6118201; doi:10.7717/peerj.5352)
Supplement: Supplemental Information 3 [file peerj-06-5352-s003.pdf]

Supplementary File S2

Study Name and Year	Reason for Exclusion
Air pollution and admissions for acute lower respiratory infections in young children of Ho Chi Minh City<br>*S. Mehta et al*<br>2011	Bronchiolitis and pneumonia admissions not separated
Clinical risk factors for life-threatening lower respiratory tract infections in children: A retrospective study in an urban city in Malaysia<br>*A.M. Nathan et al*<br>2014	Pneumonia admissions as clinical outcome
Do environmental pollutants influence the onset of respiratory syncytial virus epidemics or disease severity?<br>*J. M. Bhatt et al*<br>2004	Review paper
Effect of air pollution on respiratory emergency room visits and hospital admissions<br>*S.C.L. Farhat et al*<br>2005	Asthma and bronchiolitis combined
Health effects of air pollution exposure on children and adolescents in Sao Paulo, Brazil<br>*A.L.F Braga et al*<br>2001	All acute lower respiratory infections combined
Impact of air pollution on physician office visits for common childhood conditions in Ontario, Canada<br>*L. Feldman et al*<br>2014	Air pollution sensitive conditions, does not separate out bronchiolitis visits
Ozone related respiratory morbidity in a low pollution region<br>*S. Magzamen et al*<br>2017	Combined acute lower respiratory admissions for ages 0-14 years
Part 4. Interaction between air pollution and respiratory viruses: time series study of daily mortality and hospital admission in Hong Kong<br>*C.M Wong et al*<br>2010	All ages of lower respiratory infections included

Residential proximity to large airports and potential health impacts in New York State<br>*S. Lin et al*<br>2007	Cross sectional study
Respiratory hospital admissions in young children living near metal smelters, pulp mills and oil refineries in two Canadian provinces<br>*A. Brand et al*<br>2016	Asthma and bronchiolitis combined
Seven day cumulative effects of air pollutants increase respiratory ER visits up to threefold<br>*C. Schvartsman et al*<br>2016	Lower respiratory obstructive disease in less than 5 year olds, incorrect age group and combination of diseases
Short term risk of hospitalization for asthma or bronchiolitis in children living near an aluminium smelter<br>*A.Lewin et al*<br>2013	Combined admissions for asthma and bronchiolitis
Spatial clusters of child lower respiratory illnesses associated with community level risk factors<br>*P.I. Beamer et al*<br>2016	Not specific to bronchiolitis and air pollutant chemicals
The effect of traffic related air pollution on infantile bronchiolitis and susceptibility to childhood asthma<br>*J.Y Lee et al*<br>2011	Experience of bronchiolitis, no data available
The effects of air pollution on children<br>*G. Marcer et al*<br>2000	Respiratory symptoms combined
The effects of short term exposure on hospital admissions for acute lower respiratory infections in young children of Ho Chi Minh City, Vietnam<br>*L. Ngo et al*<br>2011	All acute lower respiratory infections combined

A preliminary assessment of the role of ambient nitric oxide exposure in hospitalization with respiratory syncytial virus bronchiolitis<br>*N.I. Mohammed et al*<br>2016	Looked at Nitric Oxide only, ineligible pollutant
Air pollution and acute respiratory infections among children 0-4 years of Age: An 18 year time-series study<br>*L.A. Darrow et al*<br>2014	Bronchiolitis and bronchitis admissions combined
Air pollution and environmental tobacco smoking during infancy may increase the risk of bronchiolitis<br>*B. Kim et al*<br>2006	Incorrect ages and too retrospective
Early life exposure to outdoor air pollution and respiratory health, ear infections, and eczema in infants from the INMA study<br>*I. Aguilera et al*<br>2013	Doctor diagnosed lower respiratory infection not specific to bronchiolitis
Effects of fine particles on children's hospital admissions for respiratory health in Seville, Spain<br>*M de P. Pablo-Romero*<br>2014	Looked at city wide levels of pollution rather than to specific admission
Exposure to traffic and early life respiratory infection: a cohort study<br>*M. B. Rice*<br>2014	All acute lower respiratory infection not specific to bronchiolitis
Fine Particulate Matter Pollution linked to respiratory illness in infants and increased hospital costs<br>*P. Sheffield*<br>2011	Cross sectional study
Haze is a risk factor contributing to the rapid spread of respiratory syncytial virus in children<br>*Q. Ye*<br>2016	Examines at geographical level

Supplementary File S2

Modifiable demographic factors that differentiate bronchiolitis from pneumonia in Nepalese children less than two years – a hospital based study<br>*Malla T et al*<br>2014	General outdoor air pollution – non specific to pollutants
Modifiable risk factors associated with bronchiolitis<br>*R. Nenna et al*<br>2017	General outdoor air pollution – non specific to pollutants
Outdoor, but not indoor, nitrogen dioxide exposure is associated with persistent cough during the first year of life<br>*A. Esplugues et al*<br>2011	Looked at respiratory problems not at outcome of interest
Respiratory syncytial virus bronchiolitis, weather conditions and air pollution in an Italian urban area: An observational study<br>*R.Nenna et al*<br>2017	Association was between temperature and pollution levels in known RSV seasons
Respiratory Syncytial virus infection in infants and correlation with meteorological factors and air pollutants<br>*S. Vandini et al*<br>2013	Not examine clinical outcome
Association of acute bronchiolitis with environmental variables<br>*A. Zamorano et al*<br>2003	City wide levels of pollutants examined
Air pollution and acute respiratory diseases in children: regression analysis of morbidity data<br>*M. Biesiada et al*<br>2000	Bronchitis and bronchiolitis cases combined
Effect of air pollution upon the hospitalisation for acute lower respiratory tract infections among the Bucharest municipality's residents<br>*Ion-Nedelcu et al*<br>2008	Not age specific examines 0-14 year olds

Effect of environmental air pollutants on wheezing airways respiratory infections in emergency room<br>*F. Orazzo et al*<br>1998	Definition of bronchiolitis is for wheezing disorder
Exposure to fine particles and bronchiolitis in infants<br>*Nicolle Mir*<br>2009	Same paper as written by Karr et al in 2009 that has been included
Exposure to vehicular traffic is associated to a higher risk of hospitalisation for bronchiolitis during the first year of life<br>*M. Lanari et al*<br>2016	Vehicular traffic not specific pollutants
Impact of air pollution in paediatric consultations in primary health care: Ecological Study<br>*R. Martin Martin et al*<br>2017	Unclear definition of bronchiolitis
Influence of respiratory viruses, cold weather and air pollution in the incidence of lower respiratory tract infections in infants children<br>*L.F. Avendano et al*<br>1999	Combines respiratory syncytial virus across all ages
Relationship of hospital admissions with respiratory syncytial virus (RSV) bronchiolitis to environmental nitric oxide<br>*J.M. Bhatt et al*<br>2000	Looking at nitric oxide, incorrect pollutant
The influence of respiratory syncytial virus infections and environmental conditions on pediatric health care demand during winter-2002 in Santiago, Chile<br>*L.F. Avendano et al*<br>2003	Not look at bronchiolitis separately
