# Supplementary material for: The effect of outdoor air pollution on the risk of hospitalisation for bronchiolitis in infants: a systematic review
Source: PeerJ. 2018 Aug 28;6:e5352. doi: 10.7717/peerj.5352 (PMC6118201; doi:10.7717/peerj.5352)
Supplement: Supplemental Information 4 [file peerj-06-5352-s004.pdf]

Supplementary File S3 – Results Table of included studies

Pollutant	Study	Lag Exposure	Crude Odds Ratio	Adjusted Odds Ratio
Particulate Matter 2.5	Karr (Karr et al. 2009a)	1 month Exposure	0.93 (0.89–0.97)	0.96 (0.92-1.01)
		Lifetime	0.90 (0.84–0.97)	0.97 (0.90-1.04)
	Karr (Karr et al. 2009b)	7 days	Not stated	1.12 (0.94-1.33)
		30 day	Not stated	1.15 (0.90-1.47)
		Lifetime	Not stated	1.14 (0.88-1.46)
	Karr (Karr et al. 2006)	1-2 days	Not stated	0.96 (0.94-0.99)
		3-5 days	Not stated	0.98 (0.96-1.00)
	Karr (Karr et al. 2004)	1-2 days	0.98 (0.95-1.00)	Not stated
		3-5 days	0.98 (0.96-1.00)	Not stated
	Girguis (Girguis et al. 2017)	Lifetime	1.05 (1.02, 1.07)*	1.09 (1.05-1.13)*
	Karr (Karr et al. 2007)	Sub-chronic (30 days)	Not stated	1.09 (1.04-1.14)*
		Chronic (Lifetime)	Not stated	1.09 (1.04-1.14)*
Particulate Matter 10	Karr (Karr et al. 2009a)	1 month Exposure	0.97 (0.94–10.01)	1.00 (0.96-1.03)
		Lifetime	0.99 (0.94–1.04)	1.03 (0.98-1.08)
	Rahman (Abdul Rahman et al. 2017)	Lifetime	1.12 (1.09-1.14)*	Not stated
	Segala (Segala et al. 2008)	0-1 day	Not stated	1.01 (0.99-1.03)
		0-4 day	Not stated	1.06 (1.03-1.10)*
Nitrogen Dioxide	Karr (Karr et al. 2009a)	1 month Exposure	1.06 (1.03–1.09)*	1.11 (1.08-1.14)*
		Lifetime	1.06 (1.02–1.09)*	1.12 (1.09-1.16)*
	Karr (Karr et al. 2009b)	Lifetime	Not stated	1.00 (0.96-1.04)
	Karr (Karr et al. 2006)	1 day	Not stated	0.97 (0.95-0.99)
		4 days	Not stated	0.96 (0.94-0.99)
	Karr (Karr et al. 2004)	1 day	Not available	Not available (stated as not statistically significant association)
		4 day	Not available	Not available (stated as not statistically significant association)
	Rahman (Abdul Rahman et al. 2017)	Lifetime	1.01 (0.98-1.03)	Not stated

	Karr<br>(Karr et al. 2007)	Sub-chronic (30 day)	Not stated	1.04 (1.00-1.08)
		Chronic (Lifetime)	Not stated	1.03 (0.99-1.07)
	Segala<br>(Segala et al. 2008)	0-1 day	Not stated	1.01 (0.98-1.03)
		0-4 day	Not stated	1.04 (1.02-1.07)*
Sulphur Dioxide	Karr<br>(Karr et al. 2009a)	1 month Exposure	1.00 (0.98–1.02)	1.03 (1.01-1.05)*
		Lifetime	1.00 (0.98–1.02)	1.04 (1.01-1.06)*
	Segala<br>(Segala et al. 2008)	0-1 day	Not stated	1.10 (1.06-1.15)*
		0-4 day	Not stated	1.12 (1.07-1.16)*
Carbon Monoxide	Karr<br>(Karr et al. 2009a)	1 month Exposure	1.03 (1.00–1.06)	1.11 (1.08-1.15)*
		Lifetime	1.03 (0.99–1.07)	1.13 (1.09-1.18)*
	Karr<br>(Karr et al. 2006)	1 day	Not stated	0.99 (0.96-1.02)
		4 day	Not stated	0.97 (0.94-1.00)
	Karr<br>(Karr et al. 2004)	1 day	Not available	Not available (stated as not statistically significant association)
		4 day	Not available	Not available (stated as not statistically significant association)
	Rahman<br>(Abdul Rahman et al. 2017)	Lifetime	0.89 (0.24-3.23)	Not stated
	Karr<br>(Karr et al. 2007)	Sub-chronic (30 day)	Not stated	1.00 (0.97-1.03)
		Chronic (Lifetime)	Not stated	1.00 (0.97-1.03)
Ozone	Karr<br>(Karr et al. 2009a)	1 month Exposure	0.97 (0.94–1.01)	0.90 (0.87-0.94)
		Lifetime	0.98 (0.94–1.02)	0.89 (0.85-0.93)
	Rahman<br>(Abdul Rahman et al. 2017)	Lifetime	0.99 (0.98-1.00)	Not stated
	Karr<br>(Karr et al. 2007)	Sub-chronic (30 day)	Not stated	0.92 (0.88-0.97)
		Chronic (Lifetime)	No	0.92 (0.88-0.97)

*statistically significant

Abdul Rahman SR, Ismail SNS, Sahani M, Ramli MF, and Latif MT. 2017. A case crossover analysis of primary air pollutants association on acute respiratory infection (ARI) among children in urban region of Klang valley, Malaysia. *Annals of Tropical Medicine and Public Health* 10:44-55. 10.4103/ATMPH.ATMPH_75_17

Girguis MS, Strickland MJ, Hu XF, Liu Y, Chang HH, Belanoff C, Bartell SM, and Vieira VM. 2017. Chronic PM2.5 exposure and risk of infant bronchiolitis and otitis media clinical encounters. *International Journal of Hygiene and Environmental Health* 220:1055-1063. 10.1016/j.ijheh.2017.06.007

Karr C, Kaufman J, Lumley T, Davis R, Shepherd K, Ritz B, and Larson T. 2004. Effect of ambient air pollution on infant bronchiolitis. *Epidemiology* 15:S31-S32. 10.1097/00001648-200407000-00068

Karr C, Lumley T, Schreuder A, Davis R, Larson T, Ritz B, and Kaufman J. 2007. Effects of subchronic and chronic exposure to ambient air pollutants on infant bronchiolitis. *American Journal of Epidemiology* 165:553-560. 10.1093/aje/kwk032

Karr C, Lumley T, Shepherd K, Davis R, Larson T, Ritz B, and Kaufman J. 2006. A case-crossover study of wintertime ambient air pollution and infant bronchiolitis. *Environmental Health Perspectives* 114:277-281. 10.1289/ehp.8313

Karr CJ, Demers PA, Koehoorn MW, Lencar CC, Tamburic L, and Brauer M. 2009a. Influence of ambient air pollutant sources on clinical encounters for infant bronchiolitis. *American Journal of Respiratory and Critical Care Medicine* 180:995-1001. 10.1164/rccm.200901-0117OC

Karr CJ, Rudra CB, Miller KA, Gould TR, Larson T, Sathyanarayana S, and Koenig JQ. 2009b. Infant exposure to fine particulate matter and traffic and risk of hospitalization for RSV bronchiolitis in a region with lower ambient air pollution. *Environmental Research* 109:321-327. 10.1016/j.envres.2008.11.006

Segala C, Poizeau D, Mesbah M, Willems S, and Maidenberg M. 2008. Winter air pollution and infant bronchiolitis in Paris. *Environmental Research* 106:96-100. https://dx.doi.org/10.1016/j.envres.2007.05.003
