# Supplementary material for: The effect of outdoor air pollution on the risk of hospitalisation for bronchiolitis in infants: a systematic review
Source: PeerJ. 2018 Aug 28;6:e5352. doi: 10.7717/peerj.5352 (PMC6118201; doi:10.7717/peerj.5352)
Supplement: Supplemental Information 5 [file peerj-06-5352-s005.pdf]

Supplementary File S4. GRADE Assessments

Pollutant and exposure	Number of studies and design, and number of infants	Conclusion	Study limitations	Inconsistency	Imprecision	Grade of evidence
PM2.5 acute exposure	2 case-crossover studies and of 41,474 infants (Karr et al. 2004; Karr et al. 2006)	Does not seem to affect risk of hospitalisation	No change	No change	+1	Moderate
PM2.5 subchronic exposure	3 case-control studies including 33,394 infants (Karr et al. 2007; Karr et al. 2009a; Karr et al. 2009b)	Unclear effect on risk of hospitalisation	+1	-1	+1	Moderate
PM2.5 lifetime exposure	4 case-control studies of 52,768 infants (Girguis et al. 2017; Karr et al. 2007; Karr et al. 2009a; Karr et al. 2009b)	May increase risk of hospitalisation	+1	-1	+1	Moderate
PM10 acute exposure	1 case crossover study of 16588 infants (Segala et al. 2008)	Unclear effect on risk of hospitalisation	+1	No change	No change	Low
PM10 subchronic exposure	1 case control study of 11,675 infants (Karr et al. 2009a)	Does not seem to affect risk of hospitalisation	+1	No change	No change	Low
PM10 lifetime exposure	1 case crossover study and 1 case control study, including 17,454 infants(Abdul Rahman et al. 2017; Karr et al. 2009a)	Unclear effect on risk of hospitalisation	-1	No change	No change	Low

NO2 acute exposure	3 case-crossover studies of 58,062 infants (Karr et al. 2004; Karr et al. 2006; Segala et al. 2008)	Unclear effect on risk of hospitalisation	+1	No change	+1	Moderate
NO2 subchronic exposure	2 case-control studies of 30,270 infants (Karr et al. 2007; Karr et al. 2009a)	Unclear effect on risk of hospitalisation	+1	No change	+1	Moderate
NO2 lifetime exposure	2 case control studies and 2 case crossover studies of 39,173 infants (Abdul Rahman et al. 2017; Karr et al. 2007; Karr et al. 2009a; Karr et al. 2009b)	Unclear effect on risk of hospitalisation	-1	No change	+1	Low
SO2 acute exposure	1 case crossover study of 16,588 infants (Segala et al. 2008)	May increase risk of hospitalisation	+1	No change	No change	Low
SO2 subchronic exposure	1 case crossover study of 11,675 infants (Karr et al. 2009a)	May increase risk of hospitalisation	+1	No change	No change	Low
SO2 lifetime exposure	1 case control study of 11,675 infants (Karr et al. 2009a)	May increase risk of hospitalisation	+1	No change	No change	Low
CO acute exposure	2 case crossover study of 41,474 infants (Karr et al. 2004; Karr et al. 2006)	Does not seem to affect risk of hospitalisation	+1	No change	No change	Low

CO subchronic exposure	2 case control studies of 30,270 infants (Karr et al. 2007; Karr et al. 2009a)	Unclear effect on risk of hospitalisation	+1	No change	+1	Moderate
CO lifetime exposure	2 case control studies and 1 case crossover study of 36049 infants (Abdul Rahman et al. 2017; Karr et al. 2007; Karr et al. 2009a)	Unclear effect on risk of hospitalisation	-1	No change	+1	Low
Ozone acute exposure	No studies	No assessment can be made				
Ozone subchronic exposure	2 case control studies of 30,270 infants (Karr et al. 2007; Karr et al. 2009a)	Does not seem to increase risk of hospitalisation and may be associated with lower risk of admission	+1	No change	+1	Moderate
Ozone lifetime exposure	2 case control studies and 1 case crossover study of 36049 infants (Abdul Rahman et al. 2017; Karr et al. 2007; Karr et al. 2009a)	Does not seem to increase risk of hospitalisation and may be associated with lower risk of admission	-1	No change	+1	Low

Abdul Rahman SR, Ismail SNS, Sahani M, Ramli MF, and Latif MT. 2017. A case crossover analysis of primary air pollutants association on acute respiratory infection (ARI) among children in urban region of Klang valley, Malaysia. *Annals of Tropical Medicine and Public Health* 10:44-55. 10.4103/ATMPH.ATMPH_75_17

Girguis MS, Strickland MJ, Hu XF, Liu Y, Chang HH, Belanoff C, Bartell SM, and Vieira VM. 2017. Chronic PM2.5 exposure and risk of infant bronchiolitis and otitis media clinical encounters. *International Journal of Hygiene and Environmental Health* 220:1055-1063. 10.1016/j.ijheh.2017.06.007

Karr C, Kaufman J, Lumley T, Davis R, Shepherd K, Ritz B, and Larson T. 2004. Effect of ambient air pollution on infant bronchiolitis. *Epidemiology* 15:S31-S32. 10.1097/00001648-200407000-00068

Karr C, Lumley T, Schreuder A, Davis R, Larson T, Ritz B, and Kaufman J. 2007. Effects of subchronic and chronic exposure to ambient air pollutants on infant bronchiolitis. *American Journal of Epidemiology* 165:553-560. 10.1093/aje/kwk032

Karr C, Lumley T, Shepherd K, Davis R, Larson T, Ritz B, and Kaufman J. 2006. A case-crossover study of wintertime ambient air pollution and infant bronchiolitis. *Environmental Health Perspectives* 114:277-281. 10.1289/ehp.8313

Karr CJ, Demers PA, Koehoorn MW, Lencar CC, Tamburic L, and Brauer M. 2009a. Influence of ambient air pollutant sources on clinical encounters for infant bronchiolitis. *American Journal of Respiratory and Critical Care Medicine* 180:995-1001. 10.1164/rccm.200901-0117OC

Karr CJ, Rudra CB, Miller KA, Gould TR, Larson T, Sathyanarayana S, and Koenig JQ. 2009b. Infant exposure to fine particulate matter and traffic and risk of hospitalization for RSV bronchiolitis in a region with lower ambient air pollution. *Environmental Research* 109:321-327. 10.1016/j.envres.2008.11.006

Segala C, Poizeau D, Mesbah M, Willems S, and Maidenberg M. 2008. Winter air pollution and infant bronchiolitis in Paris. *Environmental Research* 106:96-100. https://dx.doi.org/10.1016/j.envres.2007.05.003
