# Supplementary material for: The effect of outdoor air pollution on the risk of hospitalisation for bronchiolitis in infants: a systematic review
Source: PeerJ. 2018 Aug 28;6:e5352. doi: 10.7717/peerj.5352 (PMC6118201; doi:10.7717/peerj.5352)
Supplement: Supplemental Information 6 [file peerj-06-5352-s006.docx]

This systematic review was undertaken as it is the first on this topic. No meta-analysis was conducted due to the lack of cohort studies in this area. This systematic review adds to the growing body of evidence that current air pollution guidelines are inadequate to protect against the hazardous effects of pollutants on health.
